# Neuromodulatory functions exerted by oxytocin on different populations of hippocampal neurons in rodents

**DOI:** 10.3389/fncel.2023.1082010

**Published:** 2023-02-02

**Authors:** Francesca Talpo, Paolo Spaiardi, Antonio Nicolas Castagno, Claudia Maniezzi, Francesca Raffin, Giulia Terribile, Giulio Sancini, Antonio Pisani, Gerardo Rosario Biella

**Affiliations:** ^1^Department of Biology and Biotechnology “Lazzaro Spallanzani”, University of Pavia, Pavia, Italy; ^2^Istituto Nazionale di Fisica Nucleare, Sezione di Pavia, Pavia, Italy; ^3^Department of Biotechnology and Biosciences, University of Milano-Bicocca, Milan, Italy; ^4^Department of Medicine and Surgery, University of Milano-Bicocca, Milan, Italy; ^5^Nanomedicine Center, Neuroscience Center, School of Medicine and Surgery, University of Milano-Bicocca, Milan, Italy; ^6^Department of Brain and Behavioral Sciences, University of Pavia, Pavia, Italy; ^7^Neurological Institute Foundation Casimiro Mondino (IRCCS), Pavia, Italy

**Keywords:** hippocampus, oxytocin, oxytocin receptor (OTR), neuromodulation, oxytocinergic pathways, neural circuit

## Abstract

Oxytocin (OT) is a neuropeptide widely known for its peripheral hormonal effects (i.e., parturition and lactation) and central neuromodulatory functions, related especially to social behavior and social, spatial, and episodic memory. The hippocampus is a key structure for these functions, it is innervated by oxytocinergic fibers, and contains OT receptors (OTRs). The hippocampal OTR distribution is not homogeneous among its subregions and types of neuronal cells, reflecting the specificity of oxytocin’s modulatory action. In this review, we describe the most recent discoveries in OT/OTR signaling in the hippocampus, focusing primarily on the electrophysiological oxytocinergic modulation of the OTR-expressing hippocampal neurons. We then look at the effect this modulation has on the balance of excitation/inhibition and synaptic plasticity in each hippocampal subregion. Additionally, we review OTR downstream signaling, which underlies the OT effects observed in different types of hippocampal neuron. Overall, this review comprehensively summarizes the advancements in unraveling the neuromodulatory functions exerted by OT on specific hippocampal networks.

## 1. Introduction

Oxytocin (OT) is a cross species and conserved hypothalamic hormone widely known for its peripheral hormonal effects in inducing labor and lactation (Nickerson et al., 1954; Caldeyro-Barcia and Poseiro, 1959; Brown et al., 2020). However, OT also acts as a neuromodulator in the central nervous system controlling various social and emotional forms of behavior such as attachment, social exploration, social recognition, aggression, anxiety, fear conditioning, and fear extinction (Donaldson and Young, 2008; Heinrichs et al., 2009; Matsuzaki et al., 2012; Dumais and Veenema, 2016; Olivera-Pasilio and Dabrowska, 2020). In humans, OT is involved in stress reduction, in increasing trust and altruism, and in improving an individual’s ability to interpret the mental and emotional states of others (Kosfeld et al., 2005; Ruissen and de Bruijn, 2015; Declerck et al., 2020). Moreover, the oxytocinergic system is associated with cognitive functions by taking part in the learning processes and in the formation of social, spatial, and episodic memory (Abramova et al., 2020). Accordingly, a link between deficits in the oxytocinergic system and neuropsychiatric disorders such as autism, schizophrenia, depression, bipolar disorder, and borderline personality disorder has been proposed (Dumais and Veenema, 2016; Iovino et al., 2018).

The hippocampus is a key structure for most of these emotional, behavioral, and cognitive functions (Sweatt, 2004; Immordino-Yang and Singh, 2013; Rubin et al., 2014), it receives input from hypothalamic OT-expressing fibers, and subgroups of hippocampal cells expresses OT receptors. OT influences hippocampus at multiple levels by modulating processes of learning and memory (Chini et al., 2014; Rajamani et al., 2018; Pekarek et al., 2020) and social behavior (Raam et al., 2017; Bertoni et al., 2021), remodeling the intrinsic hippocampal circuit in newborn autism models (Raam et al., 2017; Bertoni et al., 2021), and stimulating adult hippocampal neurogenesis (Leuner et al., 2012; Lin et al., 2017). The action of OT in the hippocampus relies on a precise, finely tuned, and timely modulation of specific neurons in each hippocampal subregion. The expression of oxytocin receptors (OTRs) is indeed restricted to specific classes of hippocampal neurons that have been determined in multiple studies (Campbell et al., 2009; Mitre et al., 2016; Lin et al., 2017, 2018; Raam et al., 2017; Lin and Hsu, 2018; Tirko et al., 2018; Cilz et al., 2019; Meyer et al., 2020; Bertoni et al., 2021). Moreover, the effects of the OT on individual OTR-expressing neurons, as well as the intracellular signaling pathways that are activated, are highly heterogeneous due to a promiscuous G protein coupling of the OTR (Gravati et al., 2010). Previous studies have described in detail the functional modulation carried out by OT on the specific neuronal types in each hippocampal region (Mühlethaler and Dreifuss, 1983; Mühlethaler et al., 1984; Raggenbass et al., 1989; Zaninetti and Raggenbass, 2000; Raggenbass, 2001; Succu et al., 2011; Owen et al., 2013; Harden and Frazier, 2016; Tirko et al., 2018; Maniezzi et al., 2019; Hu et al., 2021), and the initial focus of this review is to summarize the main findings of these studies. Since excitatory and inhibitory hippocampal neurons of each subregion are differently modulated by OT, the release of OT in hippocampal networks could result in a transient reshaping of the excitation/inhibition (E/I) balance. These changes could in turn affect signal processing and the subsequent information transfer throughout the hippocampal network. A similar oxytocinergic neuromodulation of E/I balance has also been demonstrated at cortical level, where it enables maternal behavior (Marlin et al., 2015), suggesting that OT could act in multiple brain areas with an apparently common general mechanism (Lopatina et al., 2018).

In this review we look at how OT is able to exert specific actions in the different regions of the hippocampal formation. Specifically, we present and discuss recent advances in our understanding on how OT modulates OTR-expressing hippocampal neurons by causing a temporary E/I balance shift that could influence signal processing and transfer through the hippocampal tri-synaptic circuit. We focus on the description of (i) the OTR-expressing neuronal populations in each hippocampal subregion, (ii) the OT-dependent electrophysiological modulation of each hippocampal neuronal population, (iii) the OT-mediated synaptic plasticity at the hippocampal synapses, (iv) the OTR signaling pathways underlying the functional effects observed on each neuronal population in the hippocampus. In this review, the systematic description of the various neuromodulatory effects, and related mechanisms, induced by OT at the hippocampal level, will provide a comprehensive neurobiological interpretation of those behavioral responses that are known to be controlled and regulated by this neuropeptide.

## 2. Oxytocin receptor distribution in the hippocampus

Oxytocin is synthesized principally in magnocellular and parvocellular neurons of the paraventricular nucleus (PVN) and in magnocellular neurons of the supraoptic nucleus (SON) of the hypothalamus (Swanson and Sawchenko, 1983). A subset of oxytocinergic neurons has also been found in the extended amygdala, and in the accessory and tuberal nuclei of the hypothalamus in mice (Biag et al., 2012; Son et al., 2022). SON and PVN magnocellular neurons mainly project to the posterior lobe of the pituitary gland (neurohypophysis) from which OT is secreted into the bloodstream (Kiss and Mikkelsen, 2005), but they also have axonal collaterals innervating numerous forebrain structures (Ross and Young, 2009; Knobloch et al., 2012). Usually, SON and PVN magnocellular neurons discharge asynchronously. However, under specific conditions (i.e., during suckling and lactation), their firing patterns become synchronized, resulting in a coordinated release of large amount of oxytocin into the bloodstream. Such synchronization is made possible by the fact that oxytocinergic magnocellular neurons regulate their own firing by somato-dendritic release of OT (Pow and Morris, 1989; Leng et al., 2008; Rossoni et al., 2008; Numan and Young, 2016; Jurek and Neumann, 2018; Valtcheva and Froemke, 2019). PVN parvocellular neurons send their axons and release the hormone in various brain areas involved in cognition, brain homeostasis, and somatic visceral responses, such as thalamus, cortex, amygdala, striatum, hippocampus (Gimpl and Fahrenholz, 2001; Stoop, 2012; Mitre et al., 2018; Son et al., 2022).

The action of OT is implemented by its binding to specific and highly conserved G-protein coupled receptors. There is only a single type of oxytocin receptor (OTR), that is able to perform a range of functions by coupling to different G-proteins (G_*q*_, G_*o*_/G_*i*_, or G_*s*_) and activating a variety of intracellular pathways (Alberi et al., 1997; Chini et al., 2008; Gravati et al., 2010; Busnelli et al., 2012; Busnelli and Chini, 2018). Moreover, the expression of OTRs is not ubiquitous, but is restricted to specific classes of neurons that vary from region to region in the brain, which leads to the generation of highly specific responses (Campbell et al., 2009; Grinevich et al., 2016; Mitre et al., 2016; Newmaster et al., 2020; Froemke and Young, 2021; Grinevich and Ludwig, 2021). The distribution of OTRs in the brain and peripheral structures has been characterized using radioligand binding, RNA *in situ* hybridization, and more recently by specific antibodies for mouse OTR (Mitre et al., 2016) and fluorescent reporter mouse lines (Yoshida et al., 2009; Nakajima et al., 2014).

In the hippocampus, the OTRs are highly expressed (Mitre et al., 2016), but they are not evenly distributed. Differences in receptor expression are observed in relation both to the hippocampal subregions and to different neuronal subtypes (Table 1). In the dentate gyrus (DG), OTRs are present almost exclusively in the region of hilus, where they are expressed by the GABAergic interneurons (INs) and, to a lesser extent, by the excitatory mossy cells (Lin et al., 2017; Raam et al., 2017; Lin and Hsu, 2018; Meyer et al., 2020; Bertoni et al., 2021). GABAergic INs of the hilus constitute a neurochemically (Lin et al., 2017; Raam et al., 2017; Meyer et al., 2020) and electrophysiologically (Meyer et al., 2020) heterogeneous population: immunohistochemical studies have shown OTR expression in somatostatin- (SST^+^), parvalbumin- (PV^+^), calretinin- (CR^+^), neuronal nitric oxide synthase- (nNOS^+^), and neuropeptide Y-positive (NPY^+^) GABAergic INs (Lin et al., 2017; Raam et al., 2017; Meyer et al., 2020), but there is evidence for OTR expression even in parvalbumin- (PV^–^) and cholecystokinin-negative (CCK^–^) INs (Harden and Frazier, 2016). In CA2/CA3 regions of the hippocampus, OTRs have been identified in both glutamatergic pyramidal cells (PYRs) and INs (Lin et al., 2017, 2018; Raam et al., 2017; Lin and Hsu, 2018; Tirko et al., 2018; Bertoni et al., 2021). Among the GABAergic OTR- positive (OTR^+^) INs of the CA2/CA3 areas those co-expressing PV are significantly more abundant than those expressing SST (Raam et al., 2017). Notably, as the CA2 subregion is related to maternal behavior, and oxytocinergic modulation and plasticity in CA2 are salient for the identification of pup vocalizations, scents and location, murine mothers show a higher percentage of OTR^+^ cells in this region compared to males and virgin females (Mitre et al., 2016). OTRs have also been identified in the CA1 region of the hippocampus (Raggenbass et al., 1989; Campbell et al., 2009; Mitre et al., 2016; Raam et al., 2017; Lin and Hsu, 2018), where OTR expression has been reported principally in GABAergic INs (Raam et al., 2017) as confirmed by functional investigations (Zaninetti and Raggenbass, 2000; Owen et al., 2013; Maniezzi et al., 2019). Finally, autoradiography experiments found OT-binding sites in the subiculum (Raggenbass et al., 1989), while more recent electrophysiological studies demonstrated a direct OTR-dependent action on subicular PYRs (Hu et al., 2021).

**TABLE 1:** Oxytocin receptor expression in hippocampal areas and neuronal phenotypes.

Region	OTR-expressing neurons	References
DG	• Hilar GABAergic INs	Campbell et al., 2009
	• (SST+, PV+, CR+, nNOs+, PV- and CCK-)	Mitre et al., 2016
	• Hilar Mossy cells (few)	Harden and Frazier, 2016
		Lin et al., 2017
		Raam et al., 2017
		Lin and Hsu, 2018
		Meyer et al., 2020
		Bertoni et al., 2021
CA2-CA3	• PV+ (principally) and SST+ GABAergic INs	Campbell et al., 2009
	• PYRs	Mitre et al., 2016
		Lin et al., 2017
		Raam et al., 2017
		Lin et al., 2018
		Lin and Hsu, 2018
		Tirko et al., 2018
		Bertoni et al., 2021
CA1	• PV+ GABAergic INs	Raggenbass et al., 1989
		Zaninetti and Raggenbass, 2000
		Campbell et al., 2009
		Owen et al., 2013
		Mitre et al., 2016
		Raam et al., 2017
		Lin and Hsu, 2018
		Maniezzi et al., 2019
SUBICULUM	• PYRs	Raggenbass et al., 1989
		Hu et al., 2021

Bibliographical references for each area are reported.

Over the last 5 years, studies have reported the expression of OTRs also in glial cells (astrocytes and microglia) of the hippocampus (Havránek et al., 2017; Zhu and Tang, 2020; Althammer et al., 2022a,b; Maejima et al., 2022), as well as in other brain areas (Loth and Donaldson, 2021; Baudon et al., 2022; Gonzalez and Hammock, 2022; Knoop et al., 2022). However, the exact function of these glial OTRs remains elusive. One possibility is that they could facilitate neuromodulation *via* the release of specific gliotransmitters (Baudon et al., 2022). The presence of OTRs in astrocytes seems to be species-specific since a prominent subpopulation (40%) of astrocytes in the dorsal CA2 region of 5–7 week old rats expressed OTRs (Althammer et al., 2022a), whereas they were not present in mice (Althammer et al., 2022b). Data regarding OTR expression and function in glial cells is far from conclusive with more research being required in this area.

## 3. Oxytocinergic functional modulation of specific neurons of the hippocampus

As noted above, the expression of OTRs throughout the hippocampus is unique and heterogeneous and it is restricted to specific but different neuronal types in each region. Consequently, the oxytocinergic neuromodulation within this area is highly complex. In general, the release of oxytocin in the hippocampus reshapes the E/I balance of local circuits through two distinct but synergic mechanisms: (i) first-order direct modulation of neurons expressing OTRs; (ii) second-order indirect modulation of neurons not expressing OTRs, locally contacted by directly modulated neurons (Froemke and Young, 2021). Here, we focus on reviewing the known direct and indirect modulatory effects exerted by OT on neuronal function within each hippocampal region.

### 3.1. Dentate gyrus (DG)

The dentate gyrus (DG) represents the main entry point of information into the hippocampus *via* perforant pathway as it is the first station of the hippocampal tri-synaptic circuit. The OTRs are mainly expressed in the GABAergic INs and the excitatory mossy cells in the hilum of the DG, whereas there has been no reported expression in the granular layer (Lin et al., 2017, 2018; Raam et al., 2017; Lin and Hsu, 2018; Meyer et al., 2020). Nevertheless, the electrophysiological effects of the OT on the neurons of this area remain poorly understood.

**FIGURE 1:**
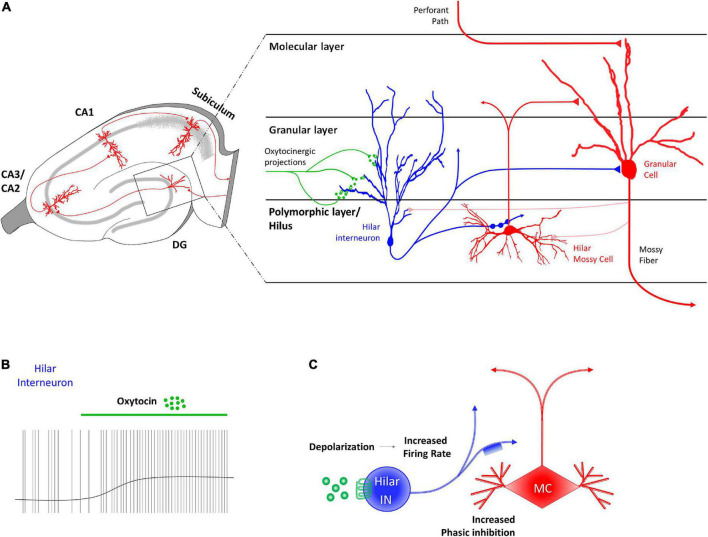
Receptor distribution, oxytocinergic projections and main effects exerted by oxytocin (OT) at dentate gyrus (DG) region of the rodent’s hippocampus. **(A)** At the DG region, OT receptors (OTRs) are found in granular layer, on the dendrites of hilar GABAergic fast-spiking interneurons (blue). Hilar GABAergic fast-spiking interneurons, whose cell body is located in the polymorphic layer, extend axonal projects both to hilar mossy cells (red) and to granular cells (red) of the granular layer. **(B)** OT induces a direct and sustained depolarization of the hilar fast spiking interneurons membrane potential and a consequent increase of their firing rate. **(C)** The OT-dependent increased firing rate of hilar fast spiking interneurons (FS IN–blue) through the binding to OTR (green) causes indirect phasic inhibition on the glutamatergic mossy cells (MC–red).

Harden and Frazier (2016) identified a subpopulation of GABAergic INs in the deep hilum of the DG of juvenile rats (Figure 1A) on which TGOT (Thr4,Gly7-oxytocin, a selective agonist of OTRs) had a direct OTR-dependent excitatory effect. The activation of the OTR in these neurons depolarized their membrane potential (probably due to an IP3-mediated increase in the intracellular Ca^2+^ and/or in the Na^+^ permeability) and increased their firing rate (Figure 1B). In turn, the activating action of TGOT on the hilar GABAergic INs was reflected in an indirect effect on the mossy cells that receive *en passant* somatodendritic synapses from the former: these cells responded to TGOT administration with an increase in the frequency and amplitude of GABAergic spontaneous inhibitory post-synaptic currents (sIPSCs) (Figures 1A, C). It is worth noting that a small subpopulation of OTR-expressing mossy cells exists (Raam et al., 2017; Meyer et al., 2020), but the direct action of OT on this neuronal type is still unknown.

The effect of OT on the local circuitry of the DG consists of an initial first-level direct activation of the hilar GABAergic INs, resulting in an increased GABA release in the hilum of the DG. This is followed by a second-level indirect inhibition of the glutamatergic mossy cells (Figure 1C). However, the final effect on the granular cells of the DG, the projective neurons of this area, remains to be investigated. Although granular cells do not express OTRs (Lin et al., 2017, 2018; Raam et al., 2017; Lin and Hsu, 2018; Meyer et al., 2020), it is reasonable to postulate that they could also be indirectly inhibited by OT, since they receive synaptic contacts from both hilar GABAergic INs (Meyer et al., 2020) and mossy cells (Scharfman, 1995; Figure 1A). The importance of the oxytocinergic modulation of the DG for the general functionality of the hippocampal circuit is highlighted in mice lacking OTRs showing an overall aberrant activation of the DG in response to a social stimulus (Raam et al., 2017). Although the exact mechanisms still needed to be addressed, it is possible to hypothesize that OT in the DG could control the progression or restriction of information flow within the hippocampal circuit (Harden and Frazier, 2016).

### 3.2. CA3/CA2 regions of the hippocampus

**FIGURE 2:**
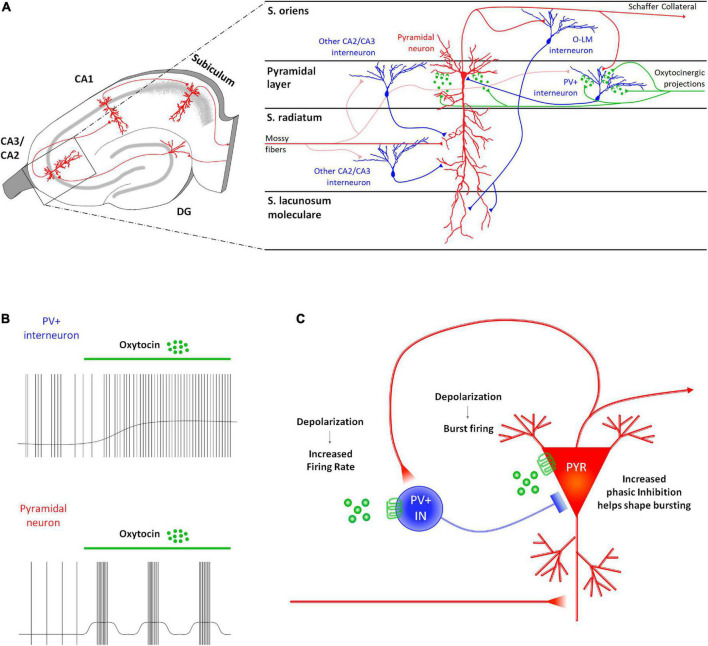
Receptor distribution, oxytocinergic projections and main effects exerted by oxytocin (OT) at CA2/CA3 (DG) region of the rodent’s hippocampus. **(A)** At the CA2/CA3 region, OTRs are found both on the projective glutamatergic pyramidal neurons (PYR–red) and on parvalbumin positive GABAergic interneurons (PV^+^ INs–blue). **(B)** In this region, OT increases the PV^+^ IN excitability by depolarizing their membrane potential and by increasing their firing rate. Also, it exerts an excitatory effect on the PYRs by depolarizing their membrane and by promoting the onset of a bursting firing pattern. **(C)** In CA2/CA3, OT induces a reciprocal modulation of the neuronal activity of PYRs and PV^+^ INs. Specifically, the OT-dependent increased excitability of the PYRs reverberates on the PV^+^ IN by increasing frequency and amplitude of the EPSPs onto the PV^+^ IN receiving Schaffer’s collaterals. In turn, OT-dependent increased excitability of PV^+^ IN supports a GABAergic input onto the PYR that modulates their burst firing and appears to be essential in promoting reliability of synaptic communication in the downstream areas of the hippocampus.

The involvement of CA3/CA2 regions in social recognition (Lu et al., 2015; Alexander et al., 2016; Raam et al., 2017; Lin et al., 2018) supports a role for oxytocinergic modulation of cellular and synaptic responses in these areas. Accordingly, CA2 and CA3 PYRs and parvalbumin positive GABAergic INs (PV^+^ INs) express OTRs in mice (Mitre et al., 2016; Figure 2A) and receive input from oxytocinergic fibers (Tirko et al., 2018). Electrophysiological characterization of the oxytocinergic modulation of CA2 has been performed by Tirko et al. (2018). They found that application of TGOT leads to increased excitability of CA2 PYRs by causing depolarization, inducing burst firing (Figure 2B), and changing the shape of the action potentials (reducing both the peak amplitude and the after-hyperpolarization). TGOT-mediated depolarization of PYRs has also been observed in the presence of glutamatergic and GABAergic blockade, indicating a first-order direct effect. Furthermore, the OTR agonist depolarizes PV^+^ INs located in the pyramidal layer causing an increase in their firing rate (Figure 2B). Since the excitability is enhanced also during synaptic blockade, the TGOT-mediated effect on PV^+^ INs is direct too. The ionic mechanism underlying these depolarizations has been proven to rely on a reduction of the M-current (Tirko et al., 2018).

During oxytocinergic modulation, PYRs and PV^+^ INs influence each other (Figure 2C). Indeed, recordings from PV^+^ INs reveal an increase in the frequency and amplitude of the excitatory post-synaptic potentials (EPSPs), while recordings from PYRs uncover an increase in the frequency and amplitude of the inhibitory post-synaptic potentials (IPSPs). The role of GABAergic input is essential during the TGOT-mediated bursts generated by PYRs: indeed, it significantly elevates the intra-burst frequency and restricts burst duration. This firing pattern is able to increase the reliability of synaptic communication (Lisman, 1997).

The TGOT-induced bursts in CA2 PYRs firing has a profound effect on the downstream CA1 area, determining the depression of both the mono-synaptic excitatory outputs and the di-synaptic inhibitory outputs toward CA1 PYRs (Tirko et al., 2018). However, the depression of inhibitory output is consistently greater than that of the excitatory output. As a result, the IPSC:EPSC ratio shifts toward a net excitatory drive, thus favoring the transmission transfer from CA2 to the downstream CA1 area (Tirko et al., 2018).

Although experimental data are still missing, authors postulated a similar mechanism also for the oxytocinergic modulation of the CA3 hippocampal region, due to the common features of these two subregions (Tirko et al., 2018).

### 3.3. CA1 region of the hippocampus

**FIGURE 3:**
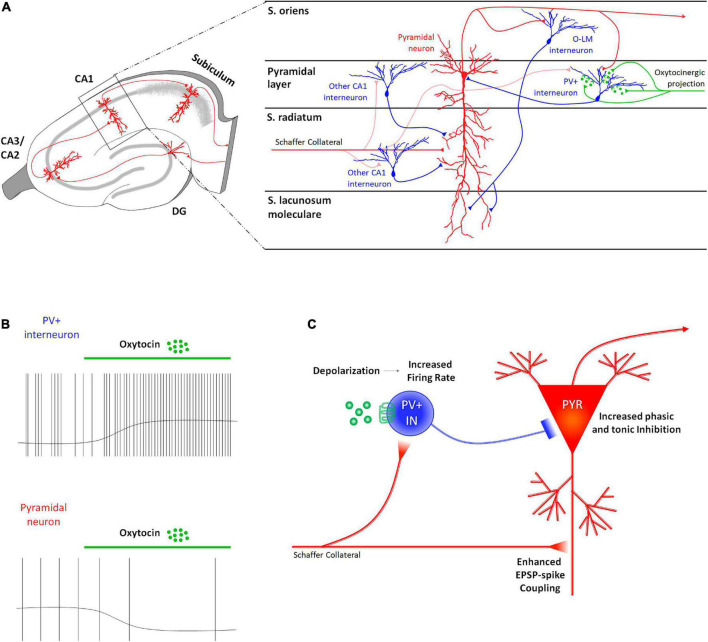
Receptor distribution, oxytocinergic projections and main effects exerted by oxytocin (OT) at CA1 region of the rodent’s hippocampus. **(A)** At the CA1 region, OTRs are expressed only by parvalbumin positive GABAergic interneurons (PV^+^ INs–blue) that receive direct oxytocinergic terminals (green) and whose cell body is located in the pyramidal layer. **(B)** In CA1, OT directly depolarizes PV^+^ INs by increasing their firing rate and indirectly (by interposition of PV^+^ INs) hyperpolarizes pyramidal neurons (PYR) by determining a reduction in their firing rate. **(C)** The OT-dependent increased excitability of the PV^+^ INs indirectly modulates the excitability of the PYRs by increasing both phasic and tonic inhibition onto them, suppressing in turn spontaneous firing of the PYRs (reduction of noise). At the same time, OT-dependent increase in the firing rate of the PV^+^ INs determines a “use-dependent reduction” of efficacy of the inhibition resulting in a more effective mono-synaptic excitatory transmission and a reduced di-synaptic inhibitory transmission arriving from Schaffer collaterals, entailing the enhancement of the EPSP-spike coupling (increase of signal). These two effects improve the fidelity and temporal precision of the information transfer through this area.

In CA1 only PV^+^ GABAergic INs express OTRs (Mitre et al., 2016; Raam et al., 2017). Accordingly, extracellular recordings performed on rats suggested that OT exerts a direct excitation on a specific population of GABAergic INs exerted by OT (Mühlethaler and Dreifuss, 1983; Mühlethaler et al., 1984; Raggenbass et al., 1989; Zaninetti and Raggenbass, 2000; Raggenbass, 2001). Consistently with these findings, OT has been found to modulate the neuronal network in the hippocampal CA1 field of the mouse by acting directly on the PV^+^ fast-spiking GABAergic INs and indirectly on the other neurons in this area, including the projecting PYRs (Figure 3A). The administration of TGOT induces a sustained depolarization of the membrane potential of the fast-spiking GABAergic INs that determines a significant increase in their firing rate when it occurs at their spike threshold (Figure 3B), as verified by two independent studies (Owen et al., 2013; Maniezzi et al., 2019). The intracellular pathways and the biophysical mechanisms at the basis of this effect are still controversial, even if the persistence of the depolarizing response in presence of synaptic blockers confirms the first-order direct nature of this effect (Maniezzi et al., 2019).

In contrast, at spike threshold PYRs are hyperpolarized by TGOT administration, and their firing rate is significantly decreased (Figure 3B) as well as their overall excitability (i.e., reduced spontaneous action potentials and decreased firing frequency in response to depolarizing injected current in TGOT vs. CTRL) (Owen et al., 2013; Maniezzi et al., 2019). Blockade of GABA_*A*_ receptors completely abolishes these effects, confirming their second-order indirect nature and their dependence on the primary direct excitation of fast-spiking GABAergic INs (Maniezzi et al., 2019). Supporting this conclusion, TGOT has been found to induce a significant increase in phasic and tonic inhibition of CA1 PYRs (Figure 3C): the increase in phasic inhibition is demonstrated by a reversible increase in sIPSCs frequency and amplitude due to enhanced release of GABA vesicles in the synaptic cleft from fast-spiking INs following TGOT administration (Owen et al., 2013; Maniezzi et al., 2019); the increase in tonic inhibition is demonstrated by the reversible slowdown in spontaneous IPSC kinetics and the outward shift in the holding current due to GABA escape from the synaptic cleft by spillover and activation of perisynaptic and extrasynaptic GABA_*A*_ receptors (Maniezzi et al., 2019).

Looking at the general impact on integration of information, signal transfer, and signal processing within the CA1 region, OT–by increasing phasic and tonic GABAergic inhibition–causes the hyperpolarization and the reduction of the excitability of the CA1 PYRs which lead to a consequent reduction in the genesis of spontaneous spikes (reduction of noise) (Figure 3C). On the other hand, the increase in the firing rate of the fast-spiking INs induces a “use-dependent reduction” in the efficacy of the inhibition of the fast-spiking INs on the PYRs (Owen et al., 2013). In these conditions inputs arriving from Schaffer’s collaterals cause more effective mono-synaptic excitatory transmission and reduced di-synaptic inhibitory transmission on PYRs, thus entailing the enhancement of the EPSP-spike coupling (increase of signal) (Figure 3C). Overall, oxytocin improves the signal-to-noise ratio in the CA1 region of the hippocampus: in this way it improves the fidelity and temporal precision of information transfer through this area.

### 3.4. Subiculum

The subiculum is the major output structure of the hippocampal formation receiving inputs from CA1 and sending outputs to many subcortical and cortical areas (Amaral and Witter, 1989; Naber and Witter, 1998; Witter, 2006). It expresses the highest density of binding sites for OT (Freund-Mercier et al., 1987; Dreifuss et al., 1989). Very little is currently known about the physiological functions and mechanisms concerning oxytocinergic neuromodulation in this brain region.

Direct OT injection into the ventral subiculum induces penile erection episodes in male rats (Succu et al., 2011), confirming the important role of this hormone for sexual behavior. The subiculum OT-induced penile erection is mediated by the activation of glutamatergic ventral neurons projecting to the ventral tegmental area (VTA) (Melis et al., 2009, 2010; Melis and Argiolas, 2011). The subiculum dependent VTA activation in turn increases the release of dopamine by mesolimbic and mesocortical dopaminergic neurons onto neurons of the nucleus accumbens and of the prelimbic medial prefrontal cortex, supporting the hypothesis that the ventral subiculum is crucial in controlling not only penile erection, but also sexual motivation, arousal, and reward (Succu et al., 2011).

**FIGURE 4:**
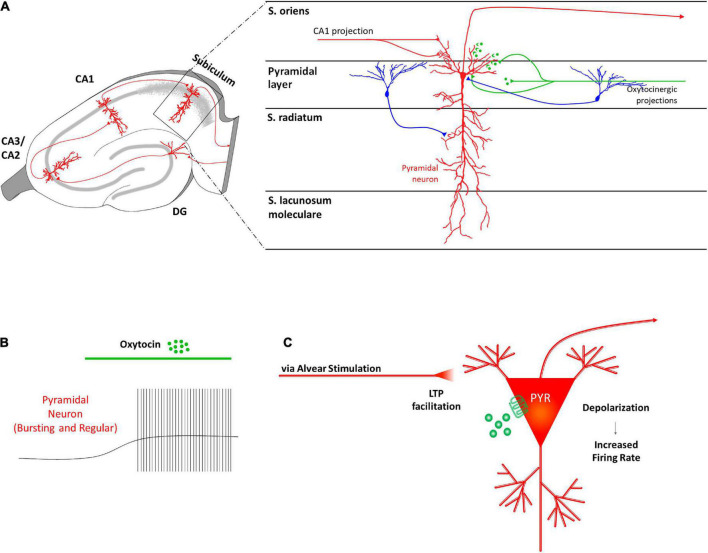
Receptor distribution, oxytocinergic projections and main effects exerted by oxytocin (OT) at subiculum region of the rodent’s hippocampus. **(A)** In the subiculum, OTRs are known to be expressed by glutamatergic pyramidal neurons (PYR–red) that receive oxytocinergic afferent fibers (green). **(B)** OT causes a significant depolarization of the membrane potential and increases the firing rate of the subicular PYRs. **(C)** The OT-dependent increased excitability of the PYRs in the subiculum is thought to be crucial in transmitting information exiting the hippocampus.

Recently, Hu et al. (2021) investigated the effects of OTR activation on the electrophysiological properties of the two types of subicular pyramidal glutamatergic neurons: intrinsically bursting cells (BCs), which are believed to contribute to synaptic plasticity, and regularly firing cells (RCs) (Cooper et al., 2005; Hu et al., 2017; Simonnet and Brecht, 2019; Figure 4A). The authors observed that OT administration directly depolarized the resting membrane potentials of both subicular BCs and RCs (Figure 4B), through two different ionic mechanisms. In about 75% of the OT-responding subicular neurons the depolarization was due to a cationic current flowing through transient receptor potential vanilloid 1 (TRPV1) channels, while in the remaining cells it is induced by the inhibition of a K^+^ current (Hu et al., 2021).

It is possible that OT-induced depolarization of the PYRs contributes to facilitate information transmission toward downstream brain areas (Figure 4C). However, how OT could affect the E/I balance in this area requires further investigation. For example, there are still no data regarding OTR expression and/or OT effect on subicular GABAergic INs although they are fundamental in the gating function exerted by the subiculum on hippocampal output activity (Benini and Avoli, 2005) and in controlling the recurrent network activity within the subicular network (Harris and Stewart, 2001; Harris et al., 2001; Witter, 2006).

## 4. Oxytocinergic modulation of hippocampal synaptic plasticity

Oxytocin serves a wide array of neuromodulatory functions in the context of synaptic plasticity in the hippocampus. It plays an important role in mediating long-term synaptic plasticity and is implicated in social, working, spatial, and episodic memory formation (Dubrovsky et al., 2002; Tomizawa et al., 2003; Lin et al., 2012, 2018; Chini et al., 2014; Park et al., 2017; Lin and Hsu, 2018; Rajamani et al., 2018; Abramova et al., 2020; Pekarek et al., 2020; Hu et al., 2021). OT application on cultured glutamatergic hippocampal neurons drives dendritic and synaptic remodeling confirming that plasticity processes occur at these synapses (Ripamonti et al., 2017). Also, OT regulates neurogenic plasticity by stimulating adult neurogenesis and by driving adult-born neural circuit integration in the hippocampus (Leuner et al., 2012; Lin et al., 2017). Systemic administration of exogenous OT in rats increases the neuronal proliferation, differentiation and complexity of dendrites on newborn neurons (Sánchez-Vidaña et al., 2016). Accordingly, when OTR is removed from the hippocampus, there is a dramatic reduction in newly generated neurons together with a marked reduction in dendritic complexity and a delay in the excitatory-to-inhibitory GABA switch (Lin et al., 2017). Thus, OT-mediated development in newborn neurons is thought to govern the response of the animal to critical social stimuli.

**FIGURE 5:**
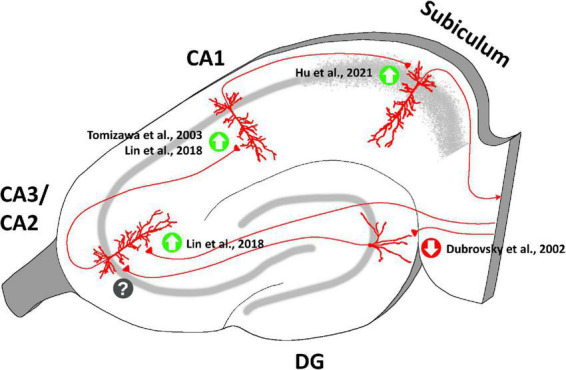
Oxytocinergic modulations of the synaptic plasticity at the different regions of the rodent’s hippocampus. OT plays a crucial role in mediating long-term plasticity at the hippocampal synapses exerting different effects in each specific hippocampal region (enhancement of long-term potentiation = green upward arrow; emergence of long-term depression = red downward arrow; unknow OT effect on plasticity = question mark). The figure shows the main bibliographic references in which the OT-dependent modulations of synaptic plasticity in the specific regions of the hippocampus have been described.

Interestingly, OT seems to exert different effects on long-term synaptic plasticity in each specific hippocampal region (Figure 5). *In vivo* experiments on anesthetized rats showed that, unexpectedly, tetanic stimulation of the perforant path following intracerebroventricular injection of OT caused long-term depression (LTD), instead of long-term potentiation (LTP), in granule cells of DG (Dubrovsky et al., 2002). A similar effect of OT was reported in the medial nucleus of the amygdala (MeA) in rats (Gur et al., 2014). On the contrary, OT signaling is necessary for LTP induction in the EC-CA2 pathway: TGOT application during high-frequency tetanic stimulation of the perforant path resulted in an acute and lasting potentiation of excitatory synaptic responses in CA2 PYRs (Lin et al., 2018). Consistently, mice with conditional deletion of hippocampal CA2/CA3 OTRs display defects in the induction of LTP in the EC-CA2 synapses (Lin et al., 2018). Despite the apparently opposite effects on synaptic potentiation, impaired oxytocinergic neuromodulation affects both of these pathways to drastically alter social recognition memory (Lin et al., 2018; Rajamani et al., 2018).

At hippocampal Schaffer’s collateral-CA1 synapses, OT enhances the ability of subthreshold synaptic stimulation to cause long-lasting LTP (Tomizawa et al., 2003; Lin et al., 2012), with the result of improving long-term spatial learning (Tomizawa et al., 2003). This result is of particular interest in light of the spatial memory enhancement that occurs in female mice during pregnancy, delivery, and lactation, when OT levels are increased both centrally and peripherally (Kinsley et al., 1999). At these synapses OT contributes to the maintenance of the late phase of LTP, but not to the early phase (Lin et al., 2012). An OT-dependent enhancement of LTP has also been reported at CA1-subiculum synapses (Hu et al., 2021).

Consistent with the central role played by OT in long-term plasticity in the hippocampus, impairments in the oxytocinergic system are associated with various pathological conditions involving deficits in learning and memory. OTR deletion in specific subregions and neurons of the hippocampus causes defects in LTP associated with hippocampal synaptic malformation and impaired long-term social recognition memory (Raam et al., 2017; Lin et al., 2018). It has been reported that an acute high-fat diet (HFD) in juvenile rats results in impaired LTP in CA1 of the dorsal hippocampus, which was related to a significant decrease in OT levels in this brain region. Systemic injection of a high dose of OT rescued HFD-induced LTP impairments (Khazen et al., 2022). Moreover, OT administration reversed synaptic plasticity impairments in other pathological conditions. Mutations in the contactin-associated protein-like 2 (CNTNAP2) are implicated in cortical dysplasia-focal epilepsy syndrome, epilepsy, attention-deficit hyperactivity disorder and autism spectrum disorders (Strauss et al., 2006; Elia et al., 2010; Mefford et al., 2010; Rodenas-Cuadrado et al., 2014). Intraperitoneal or intranasal application of OT in mice lacking CNTNAP2 transiently rescued their social behavior deficits (Peñagarikano et al., 2015). Intracerebroventricular injection of OT in a Shank3-deficient rat model of Phelan-McDermid syndrome (PMS) reduced the *in vitro* and *in vivo* synaptic plasticity impairments, partially rescuing long-term social recognition memory and attention deficits (Harony-Nicolas et al., 2015). Amyloid β peptides – that typically accumulate in the brain in Alzheimer’s Disease – strongly suppress LTP in the hippocampus, but OT administration has been found to reverse this pathological effect (Takahashi et al., 2020). Stress-induced impairments in hippocampal synaptic plasticity and spatial memory in rats can be rescued by intranasal OT administration before or after the stress event (Lee et al., 2015; Park et al., 2017).

## 5. Oxytocin-mediated intracellular signaling in the hippocampus

**FIGURE 6:**
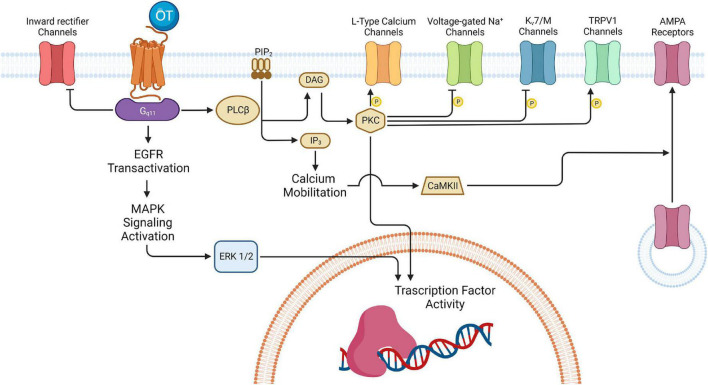
Oxytocinergic-mediated intracellular signaling in hippocampus. The various effects exerted by OT in the several regions of the hippocampus are all ascribable to the activation of the 7-transmembrane domain G protein-coupled receptors for oxytocin (OTR). In the hippocampus, OTR is usually coupled to G_q/11_ that activates various signaling cascades with different downstream effects, depending on the hippocampal region and neuronal type involved. One of the pathways activated by G_q/11_ leads to the inhibition of the inward rectifier K^+^ channels: this is one of the mechanisms possibly implicated in the depolarization of the CA1 FS-INs. Also, G_q/11_ can induce activation of the mitogen-activated protein kinase (MAPK) cascade culminating in the extracellular signal-regulated kinase 1/2 (ERK1/2) translocation to the nucleus and the activation of transcription factors involved in long-term plasticity processes. Besides, G_q/11_ determines activation of phospholipase C β (PLCβ) which leads to protein kinase C (PKC) activation and the subsequent phosphorylation of different targets (i.e., L-Type Ca^2+^ channels in CA1 GABAergic interneurons, voltage-gated Na^+^ channels and the Kv7 channels in the PYR neurons of CA2/CA3; TRPV1channels in the PYR of the Subiculum). In some cases, PKC can also activate different transcription factors thus promoting gene expression. In parallel, PLC can also bring IP3-mediated increase in the intracellular Ca^2+^ which, by activating calcium/calmodulin-dependent protein kinase II (CAMKII), promotes α-amino-3-hydroxy-5-methyl-4-isoxazolepropionic acid receptor (AMPAR) recruitment at the entorhinal (EC)-CA2 synapses to support synaptic potentiation. DAG, diacylglycerol; IP_3_, inositol 1,4,5-trisphosphate; PIP_2_, phosphatidylinositol 4,5-bisphosphate. Created with BioRender.com.

The action of OT in the brain is conveyed through the OTRs, which are 7-transmembrane domain G protein-coupled receptors that can activate different intracellular signaling pathways depending on their coupling to G_q_, G_o_/G_i_, or G_s_ proteins (Alberi et al., 1997; Gravati et al., 2010; Busnelli et al., 2012; Busnelli and Chini, 2018). It has been demonstrated that different OT concentrations determine the specific coupling of OTRs to different G_q_ and G_o_/G_i_ protein subtypes (Busnelli et al., 2012). In the hippocampus, OTRs seem to be coupled to G_q/11_ proteins (Lin and Hsu, 2018; Cilz et al., 2019; Froemke and Young, 2021) that can activate multiple signaling cascades with a variety of downstream effects (Figure 6).

The principal OTR signaling cascade involves a G-protein mediated increase in the activity of phospholipase C β (PLCβ), which in turn hydrolyses phosphatidylinositol 4,5-bisphosphate (PIP2) to generate inositol 1,4,5-trisphosphate (IP3) and diacylglycerol (DAG). IP3 increases intracellular Ca^2+^ release and DAG activates protein kinase C (PKC). The increase in intracellular Ca^2+^ together with the phosphorylation of various receptors and/or ion-channels are at the basis of the depolarizing effects identified in different populations of hippocampal neurons. The G_q/11_ pathway also transactivates epidermal growth factor receptors (EGFRs) and the mitogen-activated protein kinase (MAPK) cascade that culminates in the extracellular signal-regulated kinase 1/2 (ERK1/2) translocation to the nucleus and the activation of transcription factors involved in long-term plasticity processes (Tomizawa et al., 2003).

Specifically, the direct depolarization of the hilar GABAergic INs of the DG has been proposed to be due to an IP3-mediated increase in the intracellular Ca^2+^ and/or an increase in the sodium permeability mediated by IP3-DAG cascade (Harden and Frazier, 2016). In parallel, an OT-induced inhibition of the enzymes of the (Ca^2+^ + Mg^2+^) ATPase family complex (Soloff and Sweet, 1982) probably underlies the LTD in the EC-DG pathway by limiting the availability of the phosphate groups required for LTP induction at these synapses (Dubrovsky et al., 2002).

The ionic mechanism underlying the direct depolarization of CA2 OT-responding neurons has been proven to rely on a reduction of the M-current mediated by the PIP2 cascade, whereas the mechanism underlying the modulation of spike shape in the same neurons is determined by the PKC-dependent phosphorylation of the Na^+^-channels that are responsible for the action potential generation (Tirko et al., 2018). In parallel, the increase of the intracellular Ca^2+^, synergistically with N-methyl-D-aspartate receptors (NMDARs) opening, determines the activation of calcium/calmodulin-dependent protein kinase II (CAMKII) which promotes α-amino-3-hydroxy-5-methyl-4-isoxazolepropionic acid receptor (AMPAR) recruitment in the EC-CA2 synapses to support synaptic potentiation (Lin et al., 2018). Interestingly, a similar mechanism of synaptic potentiation–modulated by arginin-vasopressin and involving activation of NMDARs and CaMKII–has also been described at Schaffer collateral-CA2 synapses (Pagani et al., 2015).

The mechanisms of OT-induced depolarization of the CA1 GABAergic INs are controversial. On one hand, Maniezzi et al. (2019) describe an up-modulation of L-type Ca^2+^ channels mediated by TGOT application. On the other hand, Owen et al. (2013) confirm the involvement of a G protein mediated modulation operated by OT, but suggest a calcium-independent mechanism. This shows that further studies are required to dissect the exact processes underlying the OT-induced depolarization of FS-INs in this area are needed. Maniezzi et al. (2019) speculate that multiple mechanisms may be involved (i.e., upregulation of L-type Ca^2+^ channels, modulation of P/Q-type calcium Ca^2+^ channels that are selectively expressed by FS-INs, inhibition of inward rectifier potassium channels, etc.), which converge the same final depolarizing effect. It is instead established that OT-induced LTP at hippocampal Schaffer collateral-CA1 synapses involves the activation of an EGFR-mediated pathway (Tomizawa et al., 2003; Lin et al., 2012). This pathway determines–through the activation of ERK1/2 signaling–the translation of an atypical PKC isoform, the protein kinase Mζ (PKMζ) (Lin et al., 2012), that has been found to be both necessary and sufficient for the maintenance of LTP and memory storage in the hippocampus (Sacktor et al., 1993; Ling et al., 2002; Pastalkova et al., 2006; Serrano et al., 2008; Hardt et al., 2010).

In the subiculum, two different ionic mechanisms underlie the OTR-induced depolarization. In most of the subicular neurons (∼75%), OTR-mediated depolarization derives from PKC-dependent activation of a cation conductance flowing through TRPV1 channels, while in a small population of subicular neurons (∼25%) it is induced by inhibition of a K^+^ current (Hu et al., 2021). The OTR-dependent depolarization is required to induce synaptic plasticity in the CA1-subiculum synapses. Two possible mechanisms to explain OTR-elicited augmentation of LTP have been proposed. Since LTP at the CA1-subiculum synapses is NMDA dependent (Wozny et al., 2008), OTR-induced depolarization facilitates NMDA receptor opening and thus augments LTP. In parallel, since TRPV1 are Ca^2+^ permeant channels, OTR-elicited activation of TRPV1 channels increases intracellular Ca^2+^, thus inducing LTP (Hu et al., 2021).

## 6. Conclusion and open questions

The hippocampus is a key structure for learning, memory formation, and spatial navigation. Furthermore, it is essential for processing information that regulates social behaviors in primates and other mammals (Maaswinkel et al., 1997; Rubin et al., 2014). As in other brain regions, the hippocampus is highly modulated by multiple neurotransmitters and peptides. Among them, OT has been found to play a primary role in regulating neuronal activity in the different anatomical districts of the hippocampus, thus providing a possible neurobiological correlate for the origin of some of the functions located in this area.

In the last 10 years considerable progress has been made in unraveling the neuromodulatory functions exerted by OT on the specific hippocampal neurons and networks, that have been comprehensively summarized in this review. Although the stimulation of OTRs can lead to the activation of multiple intracellular signaling cascades, with different downstream effects depending on the hippocampal region and neuronal type involved, their overall results are (i) improvement of long-term plasticity at hippocampal synapses and (ii) facilitation of signal processing and the fidelity of information transfer through the stations of the hippocampal tri-synaptic circuit. These effects exerted by OT on hippocampal neuronal networks likely underlie many neuromodulatory functions of this peptide on specific aspects of important cognitive and behavioral phenomena such as sexual receptivity, maternal attachment, stress and fear extinction, social exploration, recognition, and memory. However, the hippocampus is not a homogenous functional unit since differences in anatomy, connectivity, and gene expression exist between the dorsal and ventral hippocampus. In particular, the dorsal region is principally involved in cognitive and memory processes whereas the ventral region is manly involved in emotional functions (Moser and Moser, 1998; Fanselow and Dong, 2010; Bannerman et al., 2014). A difference in OTR density was observed between the ventral and dorsal hippocampus, with the former presenting a higher number of OTR-expressing cells than the latter (Lin and Hsu, 2018). These differences suggest a possible differential neuromodulatory action exerted by OT on these two regions that deserves to be investigated further. OT seems to be involved in consolidation of social memory traces in the dorsal hippocampus (Raam et al., 2017; Lin et al., 2018; Bazaz et al., 2022), while there is a knowledge gap concerning the role of OT in ventral hippocampus and it would be worthwhile to investigate the effect on emotion and affection that this neuropeptide could have specifically in this hippocampal portion. Besides, these complex behaviors result from interactions between multiple brain areas. Despite the reported advancement, further studies are needed to deepen our understanding of the relationships between the oxytocinergic modulation of the hippocampal networks and other brain areas that express high levels of OTRs, such as amygdala or striatum. Extending knowledge in this field could be crucial in determining pharmacological targets and approaches for the treatment of neuropsychiatric disorders such as autism or schizophrenia, that have been reported to be associated with impairments in the oxytocinergic system.

Another crucial point that remains to be investigated concerns the specific contributions of different sources of endogenous OT reaching the hippocampus. OT is released in the hippocampus by the OT-producing parvocellular and magnocellular neurons of the PVN and the magnocellular neurons of the SON (Gimpl and Fahrenholz, 2001; Ross and Young, 2009; Knobloch et al., 2012; Stoop, 2012; Mitre et al., 2016, 2018; Son et al., 2022), but it is also secreted in the bloodstream by axon terminals of the magnocellular neurons of the same nuclei that reach the neurohypophysis and release OT into the neuro-hypophyseal capillaries (Kiss and Mikkelsen, 2005). OT in the blood circulation has a primarily peripheral role, especially in parturition and in lactation. However, there are pieces of evidence suggesting that OT could be re-captured at the neuronal level by crossing the blood-brain barrier thanks to the binding with a dedicated (RAGE) transporter present in endothelial cells at this interface (Yamamoto and Higashida, 2020). Verification that this mechanism exists would be of great importance for determining the relationship between the birth event and the modifications that occur in the mothers’ brain in the peri- and post-partum, which are aimed at favoring the care of the offspring and reinforcing the parental bond. In this case, indeed, not only the central release of OT, but also the reuptake from the blood could contribute to neuromodulation in new mothers. Also, OT seems to be absorbed on intestinal epithelial cells at the blood-intestinal barrier (Yamamoto and Higashida, 2020). OT passing to the milk during lactation could then be implicated in the attachment of offspring to parents, again through re-capture at neuronal level in the pups. These mechanisms could explain how several OT-related behaviors are sex-dependent (i.e., pup distress calls, somatosensory stimuli for lactation and nursing) (Donaldson and Young, 2008; Heinrichs et al., 2009; Dumais and Veenema, 2016; Brown et al., 2020), although the organization of the oxytocinergic system in the brain apparently does not vary between sexes or individuals (Knobloch et al., 2012; Son et al., 2022). Is the concentration of OT in the hippocampus and the whole brain comparable between virgin females and new mothers? Are there brain areas where the post-partum concentration of OT is higher in mothers and/or pups? Are there differences between males and females in oxytocinergic neuromodulation in specific behavioral contexts? Do different levels of OT involve the expression of different levels of OTRs? These are only a few examples of open questions in this area of research that need to be addressed. The exact course of the hypothalamic oxytocinergic projections directed to the hippocampus also remains to be investigated. Do oxytocinergic inputs to different hippocampal areas originate from a uniform neuronal population or is there a topographic organization of the inputs? In this sense, retrotracing and optogenetic experiments may help, which will also reveal other brain areas that receive simultaneous neuromodulation with the hippocampus from divergent inputs of the hypothalamus.

Finally, the interaction between OT and other neuromodulators in the hippocampus deserves more in-depth exploration. The function of the hippocampus is highly regulated by various neurotransmitters and accumulating evidence indicates that OT has the potential to interact with other neurotransmitter systems. For example, dopamine D2 receptor (D2R)/OTR heterocomplexes (de la Mora et al., 2016) and OTR/serotonin 2C receptor (5-HTR2C) heterocomplexes (Chruścicka et al., 2019, 2021) have been described in striatum and amygdala, which are brain areas functionally connected to the hippocampus. The formation of these heteroreceptors modifies the standard response to the neuromodulatory molecules, by enhancing or reducing signaling when co-release occurs. Similar mechanisms are also likely to occur in the hippocampus, where numerous dopamine and serotonin receptors are expressed, but to our knowledge they have never been investigated or described so far.

## Author contributions

GB, FT, and CM conceptualized the manuscript. GB, FT, PS, AC, CM, and FR wrote the initial manuscript and designed the figures. GB and FT critically revised the manuscript. GB and GS handled the funding. FT, PS, AC, CM, FR, GT, GS, AP, and GB contributed to the manuscript, approved the final version, and ensured that questions related to the accuracy or integrity of any part of the work are appropriately investigated and resolved. All persons designated as authors qualify for authorship and all those who qualify for authorship are listed and contributed to the article and approved the submitted version.
